# Supplementary material for: Elucidation of Novel Therapeutic Targets for Breast Cancer with ESR1-CCDC170 Fusion
Source: J Clin Med. 2021 Feb 4;10(4):582. doi: 10.3390/jcm10040582 (PMC7913953; doi:10.3390/jcm10040582)
Supplement: Supplementary file 1 [file jcm-10-00582-s001.zip › supplementary/Figure S2.pdf]

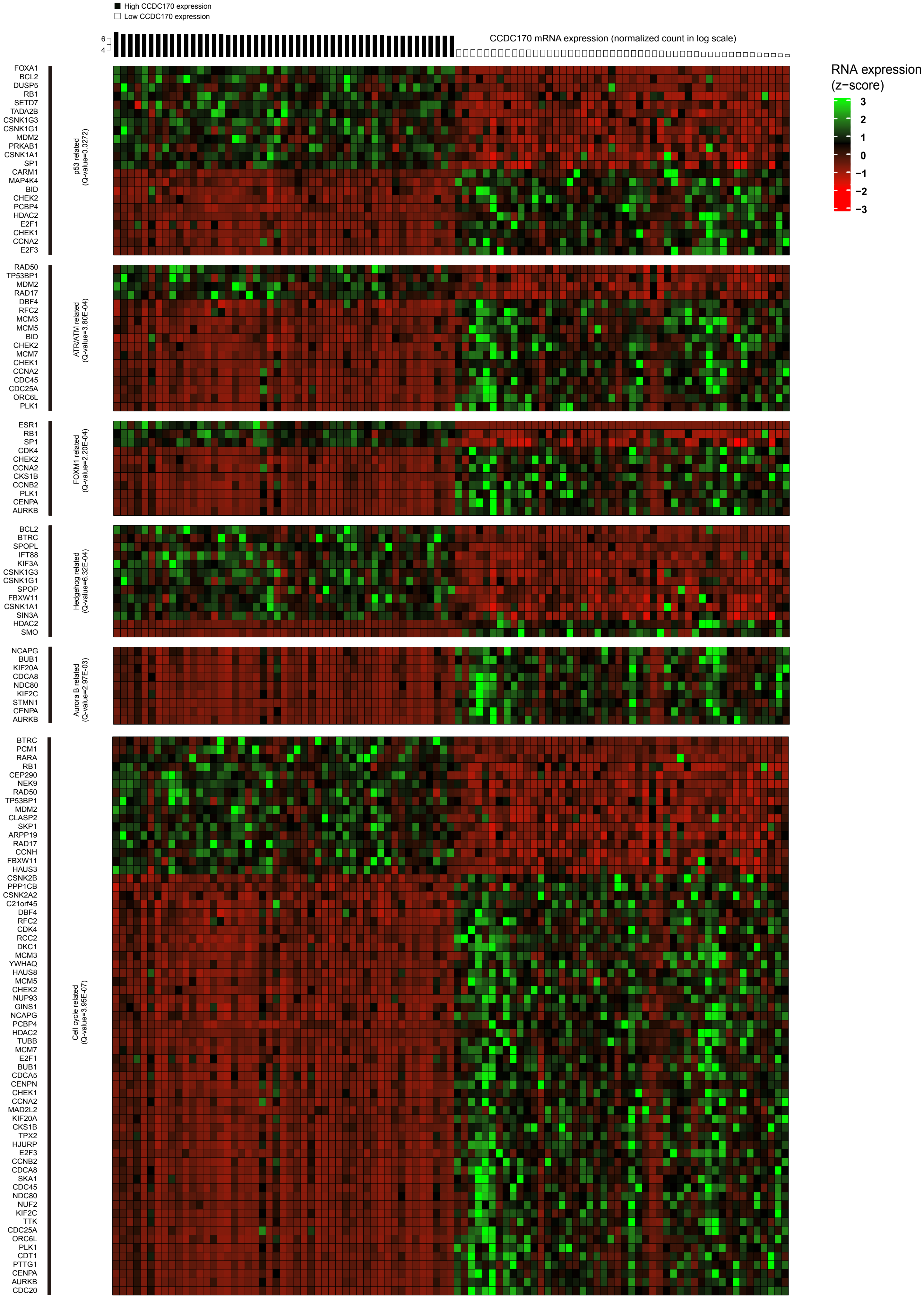
High CCDC170 expression
Low CCDC170 expression
6
4
CCDC170 mRNA expression (normalized count in log scale)
RNA expression
(z−score)
3
2
1
0
−1
−2
−3
p53 related
(Q-value=0.0272)
FOXA1
BCL2
DUSP5
RB1
SETD7
TADA2B
CSNK1G3
CSNK1G1
MDM2
PRKAB1
CSNK1A1
SP1
CARM1
MAP4K4
BID
CHEK2
PCBP4
HDAC2
E2F1
CHEK1
CCNA2
E2F3
ATR/ATM related
(Q-value=3.80E-04)
RAD50
TP53BP1
MDM2
RAD17
DBF4
RFC2
MCM3
MCM5
BID
CHEK2
MCM7
CHEK1
CCNA2
CDC45
CDC25A
ORC6L
PLK1
FOXM1 related
(Q-value=2.20E-04)
ESR1
RB1
SP1
CDK4
CHEK2
CCNA2
CKS1B
CCNB2
PLK1
CENPA
AURKB
Hedgehog related
(Q-value=6.32E-04)
BCL2
BTRC
SPOPL
IFT88
KIF3A
CSNK1G3
CSNK1G1
SPOP
FBXW11
CSNK1A1
SIN3A
HDAC2
SMO
Aurora B related
(Q-value=2.97E-03)
NCAPG
BUB1
KIF20A
CDCA8
NDC80
KIF2C
STMN1
CENPA
AURKB
Cell cycle related
(Q-value=3.95E-07)
BTRC
PCM1
RARA
RB1
CEP290
NEK9
RAD50
TP53BP1
MDM2
CLASP2
SKP1
ARPP19
RAD17
CCNH
FBXW11
HAUS3
CSNK2B
PPP1CB
CSNK2A2
C21orf45
DBF4
RFC2
CDK4
RCC2
DKC1
MCM3
YWHAQ
HAUS8
MCM5
CHEK2
NUP93
GINS1
NCAPG
PCBP4
HDAC2
TUBB
MCM7
E2F1
BUB1
CDCA5
CENPN
CHEK1
CCNA2
MAD2L2
KIF20A
CKS1B
TPX2
HJURP
E2F3
CCNB2
CDCA8
SKA1
CDC45
NDC80
NUF2
KIF2C
TTK
CDC25A
ORC6L
PLK1
CDT1
PTTG1
CENPA
AURKB
CDC20
