# Supplementary material for: Elucidation of Novel Therapeutic Targets for Breast Cancer with ESR1-CCDC170 Fusion
Source: J Clin Med. 2021 Feb 4;10(4):582. doi: 10.3390/jcm10040582 (PMC7913953; doi:10.3390/jcm10040582)
Supplement: Supplementary file 1 [file jcm-10-00582-s001.zip › supplementary/supplementay tables.pdf]

Table S1. Comparisons in clinical and pathological characteristics of CCDC170 high expression group including E:C fusion-positive cases and CCDC170 high expression group without E:C fusion.

	**High Expression including E:C Fusion-positive Cases (*N* = 48)**	**High Expression without E:C Fusion (*N* = 48)**
age	55–78	45–77
sex		
- female	48 (100.0%)	48 (100.0%)
Vital status		
- alive	41 (85.4%)	42 (87.5%)
- dead	7 (14.6%)	6 (12.5%)
Stage		
- stage I	5 (10.6%)	6 (12.8%)
- stage Ia	2 (4.3%)	2 (4.3%)
- stage Ib	1 (2.1%)	1 (2.1%)
- stage II	0 (0.0%)	0 (0.0%)
- stage IIa	20 (42.6%)	20 (42.6%)
- stage IIb	6 (12.8%)	7 (14.9%)
- stage III	0 (0.0%)	0 (0.0%)
- stage IIIa	6 (12.8%)	4 (8.5%)
- stage IIIb	3 (6.4%)	3 (6.4%)
- stage IIIc	3 (6.4%)	3 (6.4%)
- stage Iv	0 (0.0%)	0 (0.0%)
- stage x	1 (2.1%)	1 (2.1%)
ER status		
- positive	43 (97.7%)	44 (100.0%)

- negative	0 (0.0%)	0 (0.0%)
- indeterminate	1 (2.3%)	0 (0.0%)
PR status		
- positive	37 (84.1%)	39 (88.6%)
- negative	7 (15.9%)	5 (11.4%)
- indeterminate	0 (0.0%)	0 (0.0%)
HER2 IHC		
0	4 (15.4%)	4 (16.7%)
- 1+	7 (26.9%)	8 (33.3%)
- 2+	11 (42.3%)	10 (41.7%)
- 3+	4 (15.4%)	2 (8.3%)
subtype		
- Basal	0 (0.0%)	0 (0.0%)
- Her2	1 (2.1%)	0 (2.1%)
- LumA	32 (66.7%)	35 (72.9%)
- LumB	15 (31.2%)	13 (27.1%)
PIK3CA mutation	14 (31.1%)	16 (35.6%)
CDH1 mutation	3 (6.7%)	4 (8.9%)
TP53 mutation	5 (11.1%)	4 (8.9%)
BRCA1 mutation	0 (0.0%)	0 (0.0%)
BRCA2 mutation	0 (0.0%)	0 (0.0%)

Table S2. Thirty-nine genes with hits of multiple pathways.

Index	Gene	Index	Gene
1	*MDM2	21	TP53BP1
2	*AURKB	22	MCM7
3	*HDAC2	23	BUB1
4	*PLK1	24	MCM5
5	*CCNA2	25	DBF4
6	*CENPA	26	CDK4
7	*CHEK2	27	MCM3
8	*CHEK1	28	BTRC
9	*RB1	29	BID
10	CCNB2	30	NCAPG
11	FBXW11	31	ORC6L
12	CDC45	32	BCL2
13	CDCA8	33	RFC2
14	E2F3	34	NDC80
15	E2F1	35	PCBP4
16	CKS1B	36	RAD17
17	CSNK1G3	37	KIF20A
18	KIF2C	38	CSNK1G1
19	CSNK1A1	39	RAD50
20	CDC25A		

* Genes involved in more than three pathways.

Table S3. Putative target genes involved in three major pathways of CCDC170 high expression group without fusion.

**Pathway Name**	***p*-value**	***q*-value**	**Pathway Source**	**Members_Input_Overlap**	**Set Size**
Cilium Assembly	5.74E-09	1.00E-05	Reactome	AHI1, ALMS1, ARL3, ARL6, B9D2, BBS12, BBS4, CLUAP1, CP110, DCTN1, DYNC1I2, DYNC2LI1, DYNLRB2, IFT140, IFT172, IFT74, IFT88, KIAA1009, LZTFL1, NEK2, NPHP1, OFD1, PKD2, TCTN1, TCTN2, TTC8, TUBB, WDR19, WDR35	187
Integrins in angiogenesis	0.00202	0.266	PID	COL1A1, COL1A2, COL3A1, COL5A1, COL5A2, COL6A2, COL6A3, CSF1, PXN	63
Deregulation of Rab and Rab Effector Genes in Bladder Cancer	0.00492	0.266	Wikipathways	EXPH5, RPH3AL, SYTL5, TBC1D10A	16
