# Supplementary figures and images for: Elucidation of Novel Therapeutic Targets for Breast Cancer with ESR1-CCDC170 Fusion
Source: J Clin Med. 2021 Feb 4;10(4):582. doi: 10.3390/jcm10040582 (PMC7913953; doi:10.3390/jcm10040582)

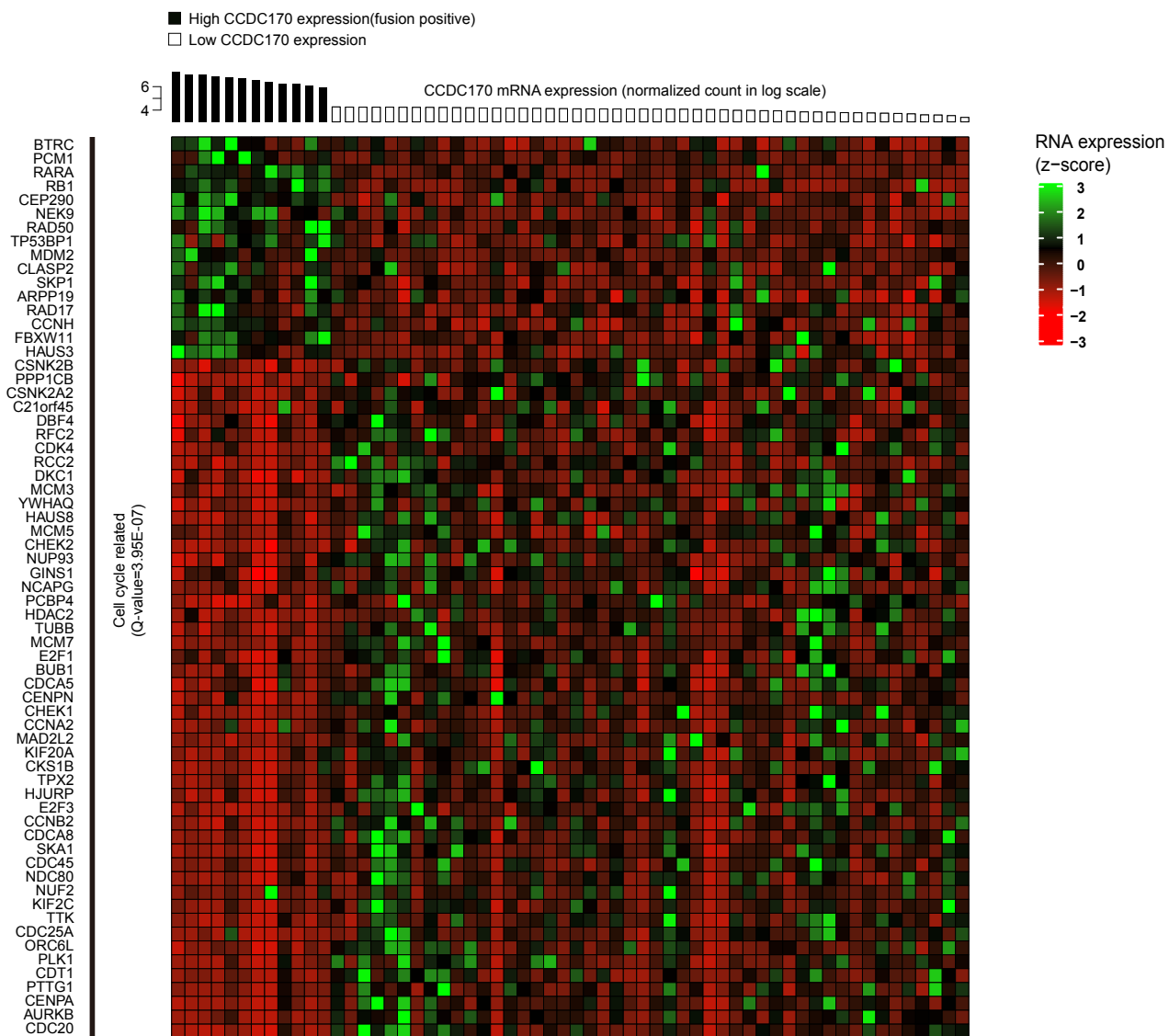

Supplement: Supplementary file 1 [file jcm-10-00582-s001.zip › supplementary/Figure S1.pdf]
